# Chronic lymphocytic leukaemia patients have a high risk of Merkel-cell polyomavirus DNA-positive Merkel-cell carcinoma

**DOI:** 10.1038/sj.bjc.6605306

**Published:** 2009-09-15

**Authors:** V Koljonen, H Kukko, E Pukkala, R Sankila, T Böhling, E Tukiainen, H Sihto, H Joensuu

**Affiliations:** 1 Department of Plastic Surgery, Helsinki University Central Hospital, Helsinki, Finland; 2 Finnish Cancer Registry, Institute for Statistical and Epidemiological Cancer Research, Helsinki, Finland; 3 School of Public Health, University of Tampere, Tampere, Finland; 4 Department of Pathology, Helsinki University and HUSLAB, Helsinki, Finland; 5 Laboratory of Molecular Oncology, Biomedicum, Helsinki, Finland; 6 Department of Oncology, Helsinki University Central Hospital, Helsinki, Finland

**Keywords:** chronic lymphatic leukaemia, Merkel-cell carcinoma, Merkel-cell polyomavirus, incidence, immunosuppression

## Abstract

**Background:**

Immunosuppression and Merkel-cell polyomavirus (MCPyV) infection may have a role in the pathogenesis of Merkel-cell carcinoma (MCC), a rare neuroendocrine carcinoma of the skin.

**Methods:**

We studied incidence of chronic lymphocytic leukaemia (CLL) and MCC from the files of the Finnish Cancer Registry and the largest hospital of Finland, Helsinki University Central Hospital, from 1979 to 2006. Presence of MCPyV DNA in MCCs was investigated by quantitative PCR.

**Results:**

We identified 4164 patients diagnosed with CLL and 172 diagnosed with MCC. Six patients diagnosed with both diseases were found; CLL was the first diagnosis in four cases and MCC in two. The standardised incidence ratio (SIR) for CLL after the diagnosis of MCC was highly elevated, 17.9 (95% confidence interval (CI), 2.2–64.6; *P*<0.001), and the SIR for MCC after the diagnosis of CLL was also elevated, 15.7 (3.2–46.0, *P*<0.01). Merkel-cell polyomavirus DNA was present in all five MCCs with tumour tissue available for analysis.

**Conclusions:**

We conclude that patients diagnosed with CLL have a substantially increased risk for MCC, and vice versa. Merkel-cell polyomavirus DNA is frequently present in MCCs that occur in CLL patients. Immunosuppression related with CLL and viral infection might explain the association between CLL and MCC.

Merkel-cell carcinoma (MCC) is a rare neuroendocrine carcinoma of the skin. In one study based on the surveillance, epidemiology, and end results (SEER) program registry of the United States, the annual incidence of MCC increased from 1.5 cases per million in 1986 to 4.4 cases per million in 2001 (Hodgson, 2005). The age-specific incidence was the highest in the elderly Caucasian population (Agelli and Clegg, 2003; Hodgson, 2005). Only a small proportion of MCCs occur before the age of 50.

Most MCCs are found in the sun-exposed skin of the head and neck or the extremities (Poulsen, 2005). Merkel-cell carcinoma usually presents with a non-tender, rapidly growing red/pink or blue/violaceous skin lesion. The median time from the lesion appearance to a biopsy is approximately 3 months (Heath *et al*, 2008). Patients diagnosed with localised, regional, and distant MCC have a 5-year relative survival of 75%, 59%, and 25%, respectively (Agelli and Clegg, 2003). Few patients diagnosed with distant metastases survive for 3 years or longer (Allen *et al*, 2005).

Merkel-cell carcinoma has been linked with immunosuppression related to various conditions, such as AIDS, organ transplantation, and presence of chronic lymphocytic leukaemia (CLL; Quaglino *et al*, 1997; Ziprin *et al*, 2000; Sinclair *et al*, 2003; Howard *et al*, 2006; Heath *et al*, 2008). The suggested association between CLL and MCC was based largely only on small single-centre series and case reports, until Howard *et al* (2006), using the SEER database, found patients diagnosed with MCC to have a standardised incidence ratio (SIR) of 2.7 for a subsequent diagnosis of CLL (95% confidence interval (CI), 0.55–7.9), and reciprocally, patients diagnosed with CLL to have a SIR of 6.9 (95% CI, 3.8 to 11.6) for a subsequent MCC. Recently, Heath *et al* (2008) suggested a considerably stronger association, estimating that CLL is more than 30-fold overrepresented among patients diagnosed with MCC based on a joint series of five institutes from Boston and Seattle.

DNA of a novel virus, named as Merkel-cell polyomavirus (MCPyV), was recently detected in the majority of MCCs investigated (Feng *et al*, 2008). The viral DNA was found in eight out of the 10 MCCs studied, and in most cases the viral DNA was integrated within the tumour genome in a clonal pattern. Patients whose MCC contained MCPyV DNA were subsequently found to have substantially more favourable survival than those whose cancer did not contain MCPyV DNA, and MCPyV-positive cancers were more frequently located in the limbs (Sihto *et al*, 2009). These clinical associations lend further support to a hypothesis that viral infection may be an early event in the molecular pathogenesis of MCC.

We investigated in the present study whether the association between CLL and MCC is reciprocal and calculated the strength of this association using the files of Finnish Cancer Registry. Nothing is known about the presence of MCPyV DNA in MCCs that occur in individuals diagnosed also with CLL. Therefore, we studied the MCCs for MCPyV DNA using quantitative PCR.

## Patients and methods

The nationwide population-based Finnish Cancer Registry, founded in 1952, covers the entire Finnish population (5.3 million in 2009). Hospitals, practicing physicians, and pathological and haematological laboratories are requested to report to the Finnish Cancer Registry all cases of cancer that come to their attention. In addition, each year, data of all death certificates that mention cancer are transferred to the Registry. Deaths and emigrations are registered in the Central Population Register and are regularly linked to the Finnish Cancer Registry. The Registry has coverage exceeding 99% (Teppo *et al*, 1994).

The study cohort includes all persons who were diagnosed with MCC in Finland from 1979 to 2006, and who were registered in the files of the Finnish Cancer Registry. All diagnoses of MCC in the Registry were based on histological examination. The follow-up for the subsequent primary neoplasm (either MCC or CLL) started from the date of the diagnosis of the first cancer (either MCC or CLL), and ended at the time of death, emigration, or the closing date of the study (December 31, 2006), whichever was earliest. The observed numbers of cancers and person–years at risk were tabulated by sex, 5-year age group, and the calendar period. The expected numbers of cancer were obtained by multiplying the stratum-specific numbers of person–years by the corresponding cancer incidence rates in Finland. The SIRs were calculated by dividing the observed numbers of cancer by the expected ones. Exact 95% CIs were calculated assuming that the numbers of observed cases followed a Poisson distribution.

Data on patient and tumour characteristics, course of the disease, treatments administered, and outcome were retrieved from hospital case records of the patients recorded as diagnosed with both MCC and CLL during their lifetime. Formalin-fixed, paraffin-embedded tissue blocks of MCC tumours were retrieved from the pathology archives. The MCC diagnoses were confirmed in a blinded manner by two researchers with a special expertise in MCC pathology (T Böhling and H Kukko). The samples were stained with haematoxylin and eosin, and we performed immunohistochemistry with antibodies for cytokeratin-20 (CK-20; DakoCytomation, Glostrup, Denmark) and thyroid transcription factor-1 (TTF-1; Novocastra, Newcastle Upon Tyne, UK) to confirm the diagnosis of MCC. For histological diagnosis of MCC, it was required for tissue morphology to be compatible with MCC in microscopy, and that the cancer cells stained positively for CK-20 and negatively for TTF-1. The longest diameter of the tumour was measured from haematoxylin and eosin-stained slides, and in case of a large tumour the diameter reported in the hospital case records was accepted. The diagnosis of CLL was based on the original analyses of the bone marrow and the peripheral blood samples, and we accepted the diagnosis of CLL as recorded in the patients’ hospital files.

Presence of MCPyV DNA was analysed from DNA extracted from representative deparaffinised tumour sections (patients 1, 2, 3, 4 and 6; Table 1) as described in a detail elsewhere (Sihto *et al*, 2009). In one case (patient 5) adequate tissue was not available for MCPyV DNA detection. Quantitation of MCPyV DNA was performed using real-time PCR with hydrolysis probes and primers specific for the viral LT3 coding region with a LightCycler 480 instrument (Roche Diagnostics GmbH, Mannheim, Germany). The relative DNA sequence copy number for each tissue sample was expressed as a ratio of MCPyV DNA-to-protein tyrosine phosphatase gamma receptor gene (*PTPRG*) DNA. Whenever MCPyV DNA was detected (i.e., when the MCPyV DNA-to-*PTPRG* DNA-ratio was greater than 0), the sample was considered positive. Each PCR product generated was treated with an ExoSAP-IT enzyme mix (product number 78201; USB Corporation, Cleveland, OH, USA) according to the manufacturer's instructions and then sequenced using BigDye3 termination chemistry and an ABI 3100 Genetic Analyzer (both from Applied Biosystems, Foster City, CA, USA). The sequences were compared with the reference sequences of MCPyV isolates obtained from the National Center for Biotechnology Information (NCBI) Entrez Nucleotide database by using LaserGene 7.2 software (DNASTAR Inc., Madison, WI, USA).

An institutional Ethics Committee approved the study protocol, and permission to analyse the tissue samples was obtained from the National Agency for Medicolegal Affairs, Finland.

## Results

### Merkel-cell carcinoma following diagnosis of CLL

According the files of the Finnish Cancer Registry, a total of 4164 individuals were diagnosed with CLL in Finland from 1979 to 2006. The follow-up yielded 19,809 person–years at risk. Altogether, in this cohort three cases of MCC were identified after the diagnosis of CLL corresponding to an SIR of 15.7 (95% CI, 3.2–46.0; *P*<0.01). The time interval from the date of diagnosis of CLL to the date of diagnosis of MCC varied from 0.2 to 9.6 years (median, 6.9 years).

In addition, one further patient diagnosed with MCC and who had CLL in history (patient no. 6; Table 1) was identified from the files of the Helsinki University Central Hospital. The patient was initially diagnosed with lymphocytic lymphoma, a disease almost identical to CLL, both morphologically and clinically. An autopsy report confirmed CLL as the underlying cause of death. This patient was not included in the calculation of the SIR.

### Chronic lymphocytic leukaemia following diagnosis of MCC

A total of 172 patients were diagnosed with MCC in Finland from 1979 to 2006, according to the files of the Finnish Cancer Registry. The follow-up yielded 713 person–years at risk. Before January 1 2007, 108 patients had died. In this cohort, two patients were diagnosed with CLL after the diagnosis of MCC, producing an SIR of 17.9 (95% CI 2.2–64.6; *P*<0.05). Both individuals were diagnosed with CLL within 1 year after diagnosis of MCC (Table 1). Search of the files of the Helsinki University Central Hospital did not identify further cases.

### Tumour characteristics and outcome

The demographic features of the patients, tumour characteristics, treatments given, and outcome data of the patients are summarised in Table 1. All patients were Caucasian, four were female, and the mean age at the time of the MCC diagnosis was 77 years (range, 63–92). Five out of the six tumours were located in an extremity. The mean tumour diameter was 1.6 cm (range, 0.4–3.0 cm). Three of the six patients had yet another malignancy besides MCC and CLL in history (breast cancer, colon cancer, and squamous-cell skin cancer, one of each).

Five of the six patients were considered to have died from CLL and none from MCC. One patient had several local recurrences of MCC, and in two cases MCC gave rise to regional lymph-node metastases in the axilla or the groin.

### Presence of MCPyV DNA in MCC

DNA for analysis of presence of MCPyV DNA was available in five out of the six MCCs detected in a patient diagnosed also with CLL. MCPyV DNA was present in all five analysed carcinomas (Table 1). The copy numbers of MCPyV DNA ranged from 0.88 to 444.4 (median, 5.49) relative to the control gene (*PTPRG*) DNA copy number. The MCPyV-positive tumours were all located in a limb.

## Discussion

The study showed remarkably strong and reciprocal association between CLL and MCC. The SIR for a diagnosis of MCC following the diagnosis of CLL was 15.7, and the SIR for the diagnosis of CLL after the diagnosis of MCC was 17.9. The two patients diagnosed with CLL following the diagnosis of MCC had the diagnosis made within 1 year from the diagnosis of MCC, suggesting that CLL may have been present already at the time of the diagnosis of MCC considering the usually long natural history of CLL.

These SIRs are in line with the results of the Boston–Seattle study, where CLL was estimated to be 30-fold overrepresented among MCC patients (Heath *et al*, 2008). Merkel-cell carcinoma is particularly common in the elderly Caucasian populations, and incidence of MCC may change substantially with time (Hodgson, 2005), which may, in part, explain differences in the ratios reported. Assuming an annual incidence of 4 per million (Hodgson, 2005), a 20- to 30-fold SIR in a patient population diagnosed with CLL translates to approximately 1 case per 10,000 patient–years.

Prolonged immunosuppression due to an underlying disease such as CLL or HIV infection, or to iatrogenic immunosuppression, likely predisposes to MCC (Penn, 1993; Manganoni *et al*, 2007). Anticancer drugs used to treat CLL, such as chlorambucil and fludarabine, may contribute to immunosuppression (Dighiero *et al*, 1998; Rashid *et al*, 2005). The link between immunosuppression and MCC may be MCPyV infection (Feng *et al*, 2008), although conclusive evidence for a causative role of MCPyV for MCC is still lacking. We recently detected MCPyV DNA in 79.8% of 114 MCCs diagnosed in Finland (Sihto *et al*, 2009), which is a slightly larger proportion than 72.3% detected in five smaller studies that included a total of 148 MCCs (Feng *et al*, 2008; Foulongne *et al*, 2008; Garneski *et al*, 2008; Kassem *et al*, 2008; Becker *et al*, 2009; Sihto *et al*, 2009). Despite the small numbers, the current finding of presence of MCPyV DNA in all MCCs among the patients who were also diagnosed with CLL suggests a role for MCPyV infection in the molecular pathogenesis of MCC in immunocompromised patients. This hypothesis is supported by the recent findings by Kassem *et al* (2009) who detected MCPyV DNA more often in Bowen's disease lesions and in basal-cell carcinomas of the skin of immunocompromised patients as compared with similar lesions detected in individuals with apparently normal immune functions.

We did not have access to tissue or blood samples to assess the presence of MCPyV in the bone marrow or in the circulating white blood cells at the time of diagnosis of CLL, which is a limitation of the study. Hypothetically, immunosuppression related to CLL or its treatments might lead to MCPyV activation and subsequent genesis of MCC, and despite the reciprocal association between CLL and MCC, MCPyV might not have a major role in the molecular pathogenesis of CLL. In line with this, MCPyV DNA was recently found to be present only rarely and at low concentrations in human haematological malignancies (Shuda *et al*, 2009). The rates of MCPyV infection may also vary in different populations (Garneski *et al*, 2009), which may influence the frequency of MCC in patients diagnosed with CLL.

We conclude that patients diagnosed with CLL have a greatly elevated risk for being diagnosed with MCC, and vice versa, as compared with the general population. Due to rarity of MCC, the absolute risk remains small, approximately 1 case per 10 000 person–years. Most MCCs diagnosed among individuals with CLL contain MCPyV DNA. A reasonable hypothesis to explain the association between MCC and CLL is immunosuppression related to CLL and/or to its treatments, which may favour development of MCCs, possibly through MCPyV infection. Clinicians who treat CLL should be aware of the greatly increased risk of MCC, because MCC can usually be cured by surgery with or without radiation therapy when detected early, whereas MCC with distant metastases is currently an invariably fatal disease.

## Figures and Tables

**Table 1:** Characteristics of patients and cancers, and clinical outcome

**Patient**	**Gender/ age** a	**Order of presentation**	**Time interval (years)**	**MCC site**	**MCC size/ regional metastases**	**Presence of MCPyV DNA in MCC**	**Treatment of MCC**	**Treatment of CLL**	**Outcome**	**Duration of follow-up (years)** b
1	F/92	MCC first	0.8	Lower arm	1.8 cm/No	Yes	Excision	None	Died of CLL	0.8
2	F/70	MCC first	0.9	Thigh	3.0 cm/No	Yes	Excision, flap, RT	Fludarabine, prednisone	Alive	5.2
3	F/63	CLL first	7.8	Upper arm	1.1 cm/No	Yes	Excision	None	Died of CLL	0.1
4	M/77	CLL first	9.6	Hand	1.0 cm/Yes	Yes	Excision	Chorambucil, prednisone	Died of CLL	2.1
5	F/81	CLL first	6.0	Chin	0.4 cm/No	NA	Excision	Fludarabine	Died of CLL	5.7
6	M/79	CLL first^c^	0.2	Thigh	2.0 cm/Yes	Yes	Excision flap	Chlorambucil	Died of CLL	0.9

Abbreviations: CLL=chronic lymphocytic leukaemia; F=female; M=male; MCC=Merkel cell carcinoma; MCPyV=Merkel-cell polyomavirus; NA=not available; RT=radiation therapy.

a At the time of diagnosis of MCC.

b Calculated from the date of diagnosis of MCC.

c The initial diagnosis was well-differentiated lymphocytic lymphoma.
